# Giant vascular eccrine spiradenoma: the first case in the scrotum and review of the literature author

**DOI:** 10.1186/s13000-021-01073-8

**Published:** 2021-04-30

**Authors:** Zan Li, Gang Li, Xin Jiang, Xiaoming Fu

**Affiliations:** 1 Department of Pathology, Chongqing General Hospital, 400013, ;2 Chongqing, China; 2 College of Stomatology, Chongqing Medical University, Songshi Bei Road 426, Yubei District, 401147 Chongqing, China

**Keywords:** Eccrine spiradenoma, Giant vascular eccrine spiradenoma, Scrotum mass, Histopathology, Immunohistochemistry

## Abstract

**Background:**

Giant vascular eccrine spiradenoma is a rare variant of eccrine spiradenoma. It is different from the eccrine spiradenoma in its larger size and greater degree of vascularity. It is often clinically confusedwith a vascular or malignant tumor.

**Case presentation:**

Here, we report a case of a 67-year-old man who presented with a tumor in the scrotum scrotal region for 6 years. The tumor had begun as a small nodule and had grown slowly with over time. Surgical excision and pathological examination revealed that the lesion was the a giant vascular eccrine spiradenoma.

**Conclusion:**

This study reports the first case of giant vascular eccrine spiradenoma in the scrotum.

## Background

Eccrine spiradenoma, which was first described in detail by Kersting and Helwig in 1956, is an uncommon benign skin adnexal tumor originating from sweat glands [1]. It commonly arises in the head, neck, trunk and extremities as a dermal or subcutaneous nodule or papule and is sometimes accompanied with pain and tenderness [2].

Giant vascular eccrine spiradenoma is a rare highly vascular variant of eccrine spiradenoma, that might be mistaken for a vascular lesion or malignant tumor due to its florid vascularity and hemorrhagic features. The gross morphology of the tumor is not distincttherefore, the clinical diagnosis of giant vascular eccrine spiradenoma is very difficult, and biopsy is required for an accurate diagnosis.

## Case presentation

### Clinical history

Our patient was a 67-year-old male who presented with a solitary mass in the scrotum. The lesion began as a small nodule that slowly increased in size over the past 6 years. The patient had no family history or traumatic history. He complained of no symptoms other than mild tenderness. Ultrasonography indicated a well-circumscribed oval mass in the dermis of the scrotum. Urological and dermatological examination revealed a single tender, skin-colored, firm dermis mass of the scrotum. The lesion was surgically excised and sent for histopathological examination.

### Pathological findings

**Fig. 1:**
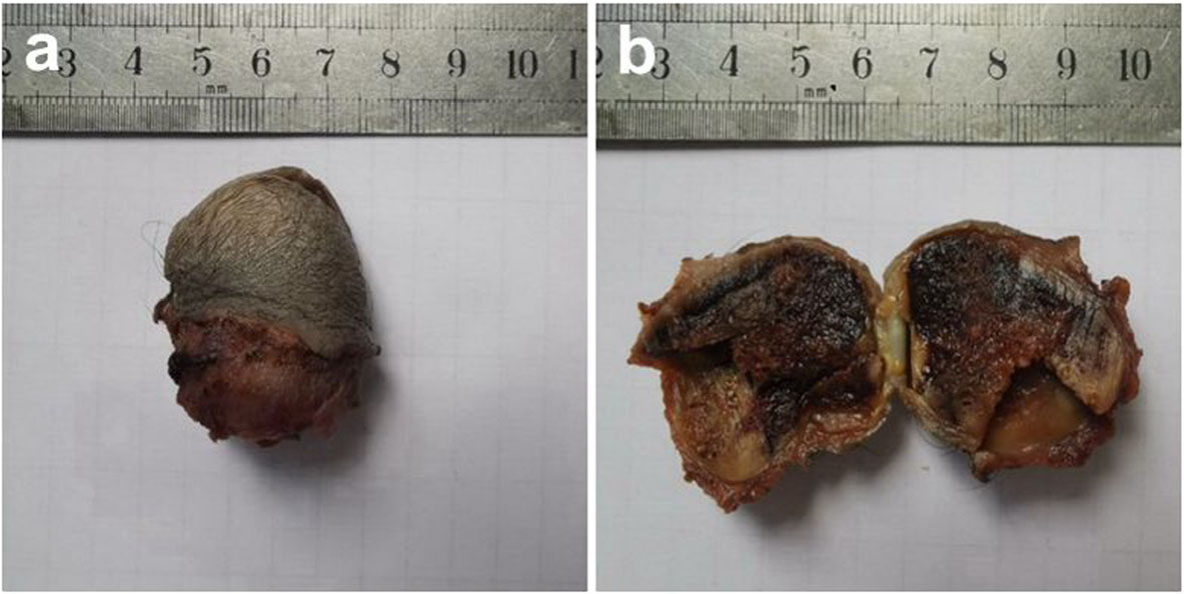
Gross examination of the resected specimen

Gross examination of the resected specimen showed that the skin surface of the scrotum was not different from other areas. The mass, which was located in the dermis of the scrotum, was approximately 3.2 × 2.3^2^ cm in size(Fig. 1a). The cut surface of the mass was hemorrhagic (Fig. 1b).

**Fig. 2:**
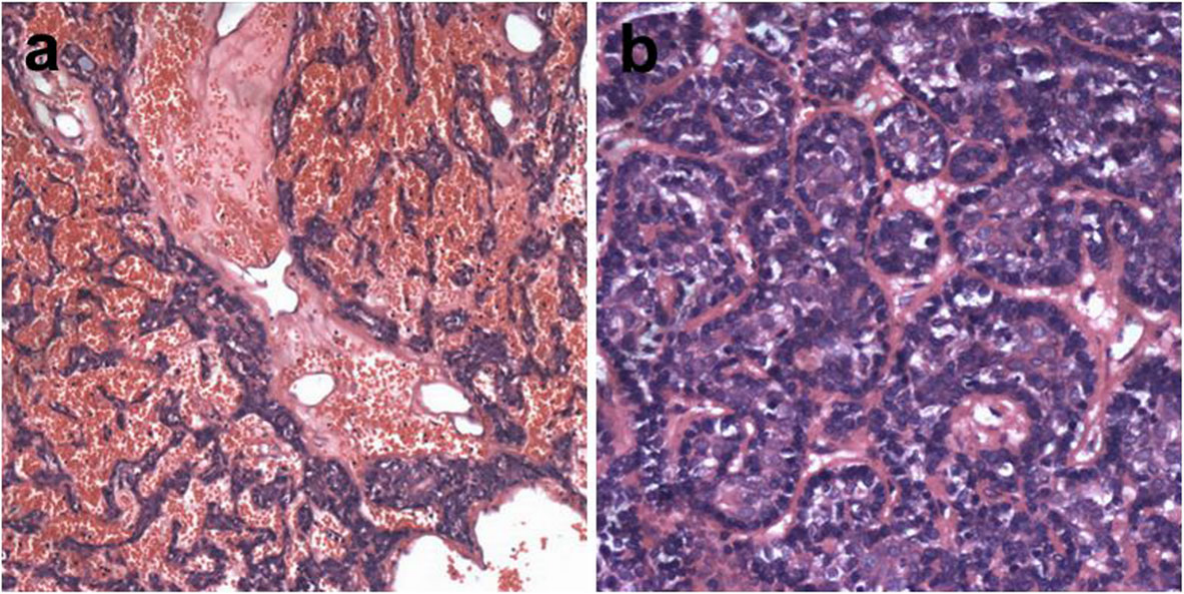
Pathologic findings (hematoxylin and eosin staining) **a** The tumor consisted of three types of cell: large pale cells situated in the center, small basaloid cells located at the periphery and scattered spindle myoepithelial cells (× 200). **b** The stroma showed numerous dilated vascular spaces containing red blood cells and extensive hemorrhage (× 100)

Histopathologic examination showed several sharply defined basophilic tumor nodules in the dermis, extending into the subcutis without any connection to the overlying epidermis. The overlying epidermis was almost intact and the surrounding connective tissue was normal. The tumor nodules comprising round-to-oval or basaloid cells arranged in acinar, sheet, cord-like, and trabecular patterns, were surrounded by a thin layer of fibrous tissue. At high magnification, three types of cells were observed in the nodules (Fig. 2a). The first were large cells with pale to slightly basophilic cytoplasm and vesicular nuclei. The nuclear membrane was thin, and the nucleolus was evident. The second were basaloid cells that surrounded the large cells. The basaloid cells were small with hyperchromatic nuclei. The third were spindle myoepithelial cells. Cytological atypia, mitoses and necrosis were not observed. Scattered lymphocytes were observed among the tumor cells and certain tubular differentiations. The stroma showed numerous dilated vascular spaces containing red blood cells and extensive hemorrhage (Fig. 2b).

**Fig. 3:**
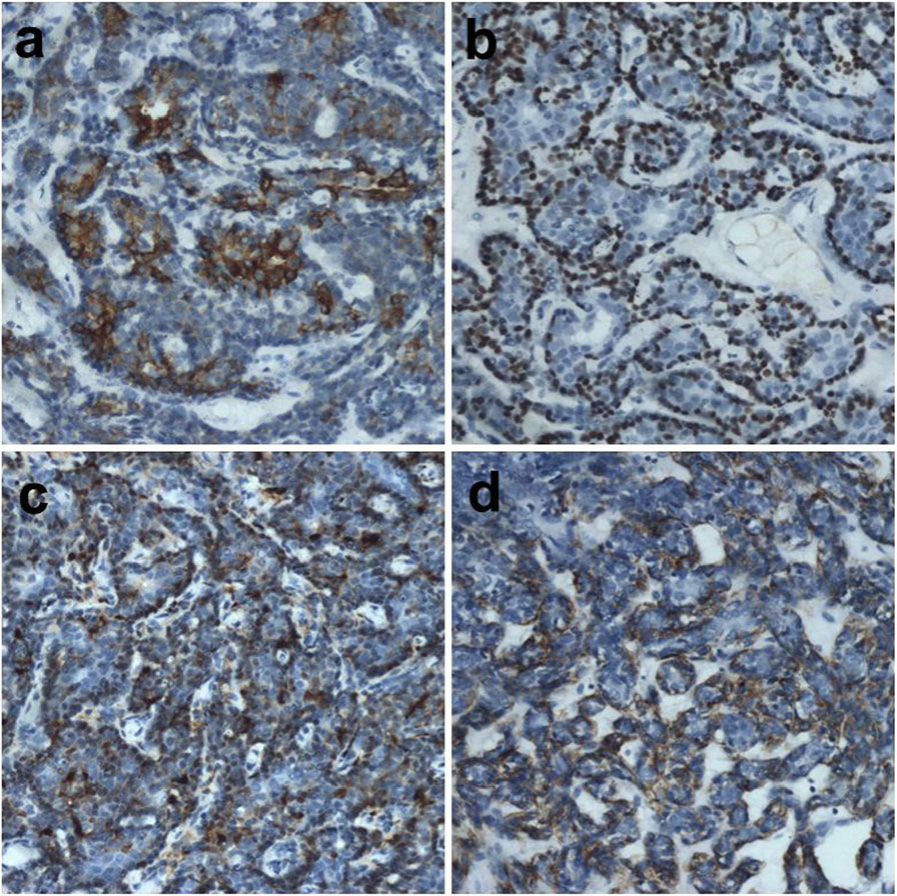
Immunohistochemical findings (**a**) cytokeratins 8 (× 200) (**b**) P63 (× 200) (**c**) S-100(× 200) (**d**) SMA (× 200)

Immunohistochemically, the large epithelial cells were strongly positive for CK, CK7 and CK8 (Fig. 3a)and negative for P63. The small basaloid cells in the outer layer were positive for P63 (Fig. 3b) and negative for SMA. The myoepithelial cells among or around the tubules were positive for S-100 (Fig. 3c) and SMA (Fig. 3d).

Thus, the diagnosis of giant vascular eccrine spiradenoma was supported by the histologic features and immunohistochemical findings.

## Discussion

Eccrine spiradenoma is a benign adnexal tumor that usually appears as a small, solitary, painful and gray -to-pink nodule and occurs equally in both males and females. Multiple eccrine spiradenoma is an extremely rare presentation comprising less than 2 % of all cases [3]. Multiple lesions have been reported to occur either in a random fashion or in linear, nevoid, blaschkoid and zosteriform patterns [4].

Lauret et al. [5] first introduced the appellation “giant eccrine spiradenoma” in 1977. Jamshidi et al. [6] reported tumors as large as 12 cm in size. Giant vascular eccrine spiradenoma, a rare variant of eccrine spiradenoma, was first described by Cotton et al. in 1986 [7]. They reported two cases of large eccrine spiradenoma with a marked degree of vascularity. The giant vascular variant is different from eccrine spiradenoma by its larger size (diameter of > 2 cm) and high vascularity. The vascularity and hemorrhagic features may result in an erroneous diagnosis by both clinicians and pathologists.

Following the two cases mentioned earlier, 12 cases of giant vascular eccrine spiradenoma have been reported in the literature. The summarization of the previously reported cases was described in Table 1. Most of the reported cases have occurred in individuals over 49 years of age; one case each occurred in men aged 41 [8] and 31 [9]. There is no significant difference in the incidence between men and women. In terms of duration, the shortest lasted 3 to 4 months, and the longest was 30 years [10]. The literature reportslesion sizes varying from 2 cm to 7 cm. The reported sites include the head, trunk and limbs. However, giant vascular eccrine spiradenoma in the scrotum has not been reported.

**Table 1:** Summary of cases of giant vascular eccrine spiradenoma

	Age/Sex	Location	Size(cm)	Duration	Symptom	Clinical Diagnosis	Unique Features
1986	74/M	Abdomen	5	2 years	Not mentioned	Angiosarcoma or Malignant melanoma	Ulcerated brown mass Extended to deep fascia
1986	84/F	Scalp	2	3–4 months	Not mentioned	Sebaceous cyst	Cystic lesion and blood-stained discharge
1988	63/F	Thigh	3.5 × 1.5	Unknown	Not mentioned	Venous thrombosis	None
1998	60/M	Hypo- chondrim	3–4	5 years	No pain	Angioma or thrombosis	Violaceous, ulcerated and bleeding lesion
2006	56/F	Back	2	3 years	Paroxysmal pain	Angiolipoma or neuroma	Erythematous to violaceous hemispheric firm nodule
2009	76/M	Shoulder	5 × 3.4 × 2.6	3 years	No pain	Not mentioned	Pale red pedunculated tumor with bleeding
2011	81/M	Forearm	1.47 × 2.57 × 2.86	3 years	Painful on palpation	Not mentioned	Blue nodule with haemorrhage
2011	49/F	Arm	2.5 × 2.5	10 years	Asymptomatic	Vascular malformation	Slightly bluish mass with the overlying intact skin
2013	52/F	Arm	3.5 × 2.2	10 years	No pain or tenderness	Calcifying epithelioma	Bluish-red nodule
2014	79/M	Chest	7 × 7 × 6	30 years	No pain or tenderness	Chronic expanding hematoma	Dark brown dome-shaped tumor
2014	31/M	Chest	2.5 × 2.5 × 1	1 years	No abnormal findings	Hemangioma	Grey brown firm nodule ulceration, bleeding
2015	54/F	Arm	2.3 × 2 × 2.6	for years	hard to palpation	Hemangioma	reddish,dome-shaped lesion, ulcerated, bleeding
2017	41/M	Leg	3.5 × 3.0	7 years	Extremely painful	Malignant tumor	Violaceus, ulcerated nodular
2018	68/M	Back	6.0 × 3.4	2 years	Slightly tenderness	Angiolipoma or cyst	Infiltrative growth pattern

Most reported giant vascular eccrine spiradenomas appeared as deep dermis or subcutaneous solitary tumors, the surface of which showed red, gray, purple, blue, or pink color. The skin over the tumor can be smooth, ulcerated or bleeding. Yamakoshi et al. [11] reported a patient with giant vascular eccrine spiradenoma on his shoulder, where there was not enough growth space and the tumor formed a giant pedunculated mass.

The composition and differentiation of giant vascular eccrine spiradenoma are stilldebated. It is generally believed that the tumors are composed of two types of cells, large pale cells situated in the center and small basaloid cells located in the periphery [12, 13]. However, some scholars have offered different conclusions. Ko et al. [14] and Corey et al. [15] described the immunohistochemical characteristics of giant vascular eccrine spiradenoma and arrived at simial conclusions. They found that giant vascular eccrine spiradenoma was typically composed of three types of cells: epithelial cells (CK+/CK7), small basal cells (p63+/SMA-), and myoepithelial cells (p63+/SMA+). The differentiation of myoepithelial cells can also be identified by positive staining for S-100 [16–18]. The results of our study are consistent with their conclusions. The immunohistological findings support the hypothesis that giant vascular eccrine spiradenoma originate from the eccrine glands and differentiates toward the secretory portion of the eccrine secretory coil [14, 15]. Electron microscopy also supports an eccrine origin for these lesions [7]. However, several authors have suggested that eccrine spiradenoma may be of apocrine origin [19, 20]. First, eccrine spiradenoma can coexist with appendage tumors, such as cylindroma, trichoepithelioma, and trichoblastoma, which are thought to originate from the apocrine gland [21, 22]. Second, eccrine spiradenoma occasionally exhibits histological features of apocrine secretion [23]. In any case, the basic theory is that the tumor originates from the secretory cells of sweat glands.

The considerable degree of vascularization is an unusual feature of giant vascular eccrine spiradenoma. At present there are two explanations for the marked vascularity. One is that giant vascular eccrine spiradenoma arises from a highly vascular region of the normal sweat gland [7, 15]. Some scholars observed an afferent vessel of the tumor with drainage into the basilic vein supplied onMRI, so they presumed the tumor was vascular in origin [24]. Another view is that the vascular component is the result of a degeneration of the tumor stroma during the process of growing and aging[25]. We are inclined to support the former view. According to previous reports, the course of giant vascular eccrine spiradenoma varies from 3 months to 30 years, and all of these cases showed high vascularization. The common type of eccrine spiradenomas lasted for 15–30 years, but no obvious vascularization was observed [26–28]. It is thus clear that the duration of the disease is not directly related to the vascularization of the tumor.

The differential diagnosis of giant vascular eccrine spiradenoma isvery important, and a variety of clinical diagnoses, including chronic expanding hematoma [10], calcifying epithelioma [29], angiolipoma or neuroma [14], desmoid [30], angiomatous lesion or thrombosis [31], angiosarcoma [7], etc., could be erroneously made.

Although malignant eccrine spiradenomas have been previously reported [32, 33], no malignant giant vascular eccrine spiradenoma has been described in the literature todate. Corey et al. [15] reported a case of giant vascular eccrine spiradenoma that showed an irregular infiltrative growth pattern into the surrounding pseudocapsule. Despite architectural atypia, they classified their case as a benign tumor because of the low proliferative index of Ki-67 staining and lack of mitotic figures. They also pointed out that the patient will be closely followed up in the future.

According to previous literature and our case, we summarized the clinical and pathological features of giant vascular eccrine spiradenoma as follows. Age: Most patients were older than 49 years, but one caseeach occurred in men aged 41 and 31. Location: All reported cases were located on the trunk or limbs except one on the scalp, and our case occurred in the scrotum. Gross appearance: The lesions presented as well-defined solitary mass larger than 2 cm in diameter and exhibited spontaneous hemorrhage and may be painful. Histological findings: Tumors usually contain three types of cells and dense dilatedvasculature.

Our patient showed no recurrence one year after the operation. It is crucial to take giant vascular eccrine spiradenoma into consideration when diagnosing scrotum subcutaneous lesions. The principle of treatment for giant vascular eccrine spiradenoma is complete surgical excision with clear margins. Postoperative follow-up is important to detect recurrence

## Conclusions

In summary, we have reported the first case of giant vascular eccrine spiradenoma in the scrotum. ITshould be taken into account in the differential diagnosis of scrotal tumors.

## Data Availability

All data generated or analyzed during this study are included in this published article.

## Abbreviations

CK7: Cytokeratin7
CK8: Cytokeratin8
ES: Eccrine spiradenoma
GVES: Giant vascular eccrine spiradenoma
HE: Hematoxylin Eosin
SMA: Smooth muscle actin
